# The mediating role of parental involvement in the relationship between social media exposure and socio-emotional development of children

**DOI:** 10.1097/MD.0000000000049932

**Published:** 2026-07-24

**Authors:** Tiruye Abdie Hussein, Wohabie Birhan Bitew, Tiruwork Tamiru Tolla

**Affiliations:** a Department of Psychology, College of Education and Behavioral Sciences, Injibara University, Injibara, Ethiopia; b Department of Psychology, College of Education and Behavioral Sciences, Bahir Dar University, Bahir Dar, Ethiopia.

**Keywords:** children, parental involvement, social media exposure, socio-emotional development

## Abstract

The study aimed to investigate the effect of parental involvement on social media exposure and socio-emotional development of children in the Awi administrative zone, Ethiopia. Data were collected in 2026 using a quantitative cross-sectional research design. Three hundred parent–child dyads (240 mother–child dyads and 60 father–child dyads) selected from diverse socioeconomic backgrounds participated in the study. The children’s ages ranged from 4 to 6 years. A survey questionnaire was employed to collect data from the parents. Data were analyzed using descriptive statistics, Pearson correlation, multiple linear regression, and the Process Procedure for Mediation, Moderation, and Conditional Process Analysis macro (model 4) for mediation analysis. The results indicated that social media exposure was significantly and negatively associated with socio-emotional development (β = −0.225, *P* < .001), while parental involvement showed a strong positive association with socio-emotional development (β = 0.666, *P* < .001), and social media exposure was negatively associated with parental involvement (β = −0.268, *P* < .001). The regression model showed that independent variables explained 53.3% of the variance in children’s socio-emotional development (*R*^2^ = 0.533, *P* < .001), indicating strong explanatory power. Mediation analysis using bootstrapping confirmed that parental involvement partially mediated the relationship between social media exposure and socio-emotional development. The indirect effect was statistically significant (β = −0.178, 95% confidence interval [−0.274, −0.078]), revealing that social media exposure influences socio-emotional outcomes both directly and indirectly through reduced parental involvement. The finding highlights parental involvement as a mediating factor in the relationship between social media exposure and children’s socio-emotional development, through which social media exposure influences the level of parental involvement, ultimately affecting socio-emotional outcomes. Strengthening active parental involvement is essential in promoting healthy developmental outcomes in early childhood within an increasingly digital environment.

## 1. Introduction

The rapid expansion of digital technology has transformed childhood experiences across the world, making social media exposure an increasingly central aspect of children’s daily lives. The widespread availability of smartphones, tablets, televisions, internet services, and social networking platforms has significantly changed the ways children learn, communicate, and interact with their environments.^[1]^ YouTube, TikTok, Instagram, and other digital applications have become common sources of entertainment, education, and social interaction for children. Social media offers opportunities for creativity, communication, and access to educational resources; growing concerns have emerged regarding its potential influence on children’s socio-emotional development.^[2]^ Socio-emotional development refers to children’s ability to regulate emotions, develop empathy, build relationships, interact socially, and manage behavioural responses effectively. These developmental competencies are essential for children’s psychological well-being, academic success and future social adjustment.^[3]^ Children exposure to screen-based social media has increased considerably over the past decade. Research conducted by^[4]^ indicated that children spend a substantial amount of time engaging with digital devices and online platforms. Studies from high- and middle-income countries have reported that average daily screen time among children continues to rise, with many children in the age range of 4 to 6 years spending several hours each day on social networking applications and digital entertainment platforms.^[5]^ Social media can facilitate learning opportunities and support communication. However, excessive and unregulated exposure has been associated with emotional difficulties, social withdrawal, behavioural problems, anxiety, and reduced interpersonal interaction.^[6,7]^ These concerns have become more evident as children increasingly replace face-to-face interaction and physical activity with screen-based engagement.

Children’s social media use is also expanding rapidly since accessibility of the internet and smartphones continue to grow in Africa. A study conducted in Ghana and South Africa demonstrates that children are increasingly exposed to social media platforms from early childhood stages.^[8]^ Similarly, in Ethiopia, children’s social media exposure has become an emerging developmental concern, particularly in urban areas where access to digital devices and internet services is growing steadily. Research conducted in Addis Ababa revealed that children spend considerable time watching television and engaging with social media, with parents expressing concerns regarding its effects on children’s behaviour and academic performance.^[9]^ A similar study conducted in Ethiopia indicated that excessive screen time is associated with behavioural difficulties, poor academic outcomes, and challenges in emotional adjustment.^[10]^ These findings imply that there is a growing need to understand how social media exposure affects children’s socio-emotional well-being within the Ethiopian context.

Children’s socio-emotional development is influenced by social interaction, emotional communication and supportive relationship with parents. Childhood is a critical developmental stage during which children learn emotional regulation, empathy, cooperation, and interpersonal skills. They learn through direct engagement with parents, peers, and their social environment. Excessive social media exposure reduces opportunities for meaningful interpersonal interaction and emotional learning.^[11]^ Studies have shown that prolonged engagement with digital media can negatively affect children’s emotional regulation, empathy, attention span, and peer relationships.^[12,13]^ Researchers have expressed concern that children who see a lot of unrealistic things may become more aggressive, anxious, and have low self-esteem.^[7]^ found that there is a growing concern about what happens to children when they use media without any rules for a long time.

Within this growing digital environment, parental involvement has been identified as an important protective factor influencing how children experience and respond to social media exposure. Parental involvement refers to parents’ active participation in monitoring, guiding, communicating and supporting children’s social media use and overall development.^[14]^ Parents who establish clear rules regarding screen time, supervise children’s online activities, discuss media content, and encourage a balanced routine could minimise negative effects associated with excessive social media exposure.^[15]^ Active parental mediation has also been associated with healthier emotional regulation, improved social competence, and better behavioural adjustment among children. In contrast, limited supervision and permissive parenting practices may increase children’s vulnerability to problematic media use and socio-emotional difficulties.^[16]^

Parental demographic characteristics such as education, income, occupation, and cultural background also influence children’s media experience and developmental outcomes. Research indicates that parents with high educational attainment are more likely to regulate children’s screen time and promote age-appropriate digital content, whereas those with limited awareness or demanding occupational responsibilities may provide less supervision.^[17]^ Besides, cultural expectations and gender-related norms shape children’s media use pattern and parental monitoring practice.^[18]^ In this regard, a study conducted in Ethiopia found that boys often receive greater freedom to access digital media, while girls are subjected to stricter parental control due to cultural expectations regarding behaviour and social interaction.^[19]^ This contextual factor demonstrates children’s social media experience is influenced not only by exposure itself but also by family environment and parental practices.

Despite the growing body of international research on social media exposure and child development, there is a scarcity of empirical study conducted on the mediating role of parental involvement in the Ethiopian context, particularly in underrepresented areas such as the Awi Zone. Previous studies conducted in Ethiopia primarily focused on the impact of media on the academic performance or behavioral outcomes of children, with limited emphasis on broader socio-emotional development and family-related influence. Furthermore, little is known about how parents in low-resource settings supervise children’s social media use, what strategies they apply, and how their involvement influences children’s emotional and social adjustment. In many Ethiopian communities, children often access media communally, share devices among family members, and engage with digital content with minimal adult supervision, creating unique developmental conditions that differ from those reported in Western society.^[20]^ Differences in cultural and socioeconomic environments limit the generalizability of these findings to Ethiopia, highlighting the need for context-specific evidence.

Therefore, the researchers believe that examining the role of parental involvement in children’s social media use and the impact on socio-emotional development is both timely and important. Understanding how parental practices mediate the effect of social media exposure may provide valuable insight into strategy that support healthy child development within a rapidly changing digital environment. This study seeks to contribute to the existing literature by exploring how social media exposure influences children’s socio-emotional development and how parental involvement shapes this relationship in the Ethiopian context. The researchers assume that the findings are expected to provide important implications for parents, educators, policymakers, and child development professionals seeking to promote responsible media use and positive socio-emotional outcomes among children.

Based on the literature reviewed, this study tested 3 hypotheses: H1: social media exposure is negatively associated with preschool children’s socio-emotional development. H2: Parental involvement is positively associated with children’s socio-emotional development. H3: parental involvement mediates the relationship between social media exposure and children’s socio-emotional development by reducing the negative effect of social media exposure.

## 2. Methods

A quantitative research design was employed to conduct the study. Data were collected from 300 parent–child dyads from 2 governmental and 2 private kindergarden (KG) schools based on sing^[21]^ sample size formula with a 5% margin of error. Table 1 shows the demographic characteristics of respondents.

**Table 1:** Demographic characteristics of respondents.

No.	Variable	Category	n	%
	Age	4 yr	28	9.3
		5 yr	101	33.7
		6 yr	171	57.0
	Sex	Male	153	51.0
		Female	147	49.0
	Grade	KG1	66	22
		KG2	84	28.0
		KG3	150	50.0
	Education level of parents	No formal education	14	4.7
		Primary education	56	18.7
		Secondary education	58	19.3
		College/university	172	57.3
	Parent occupation	Unemployed	43	14.3
		Informal employment	33	11.0
		Government worker	158	52.7
		Private worker	66	22.0
	Average income of parent in Birr	Under 3000	69	23.0
		3000–6000	33	11.0
		6001–9000	31	10.3
		9001–15,000	78	26.0
		Above 15,000	89	29.7

KG *=* kindergarten.

The age of children ranged from 4 to 6 years. The sample was comprised of 80% mothers (n = 240) and 20% fathers (n = 60). More than half of the parents have a college or university education (57.3%). A smaller proportion completed secondary education (19.3%) or primary education (18.7%), while only a few of them have no formal education (4.7%). A survey questionnaire was employed to collect data from parents. The majority of parents (52.7%) were government employees, while 22.0% of them were private sector workers. Meanwhile, a smaller proportion of them (14.3%) were unemployed, and the rest (11.0%) were engaged in informal employment. This indicates that the majority of families have stable and formal employment. The income distribution shows a mixed socioeconomic background.

The largest proportion of participants reported a monthly household income above 15,000 Ethiopian Birr (ETB) (29.7%), followed by 9001 to 15,000 ETB (26.0%) and below 3000 ETB (23.0%). A smaller proportion reported income of 3000 to 6000 ETB (11.0%) and 6001 to 9000 ETB (10.3%). Although the study was conducted in a low-resource setting, the sample included participants from diverse socioeconomic backgrounds, including relatively well-educated and middle- to higher-income households. Therefore, the term “low-resource setting” refers to the broader contextual and infrastructural environment in which the study was conducted rather than implying that all participants were from low-income or low-education households. Most of the children are 6 years old (57.0%), followed by those aged 5 years (33.7%). At the same time a smaller proportion is 4 years old (9.3%). This indicates that the sample mainly consists of older preschool children. The sex distribution is nearly equal, with 51.0% male and 49.0% female participants. In terms of the grade level of children, half of the participants were in KG3 (50.0%), followed by KG2 (28.0%) and KG1 (22.0%). After receiving ethical approval from Injibara University Institutional Research Ethics Review Committee (Ref. No. IU/IRERC/1057/26), participants were recruited using a stratified sampling technique from 4 selected schools (2 governmental and 2 private). Within each school, eligible participants were selected to ensure representation across school types. Before completing the survey, participants provided both written and oral informed consent. The survey took approximately 10 to 15 minutes to complete.

## 3. Measures

All study variables were measured using a total of 48 items structured in a questionnaire adapted from the Ages and Stages Questionnaire, 2nd Edition (ASQ-2) and the Valkenburg instrument. Each construct was assessed using multiple items rated on a 5-point Likert scale ranging from 1 = “Never” to 5 = “Always”. For each scale, item scores were averaged to compute composite variables, with higher scores indicating higher levels of the construct.

## 4. Reliability test

Table 2 shows the result of the reliability test.

**Table 2:** Reliability test result.

Reliability statistics	
Cronbach alpha	No. items
0.927	53

The reliability test conducted for the study titled “The Influence of Social Media Exposure on Children’s Socio-Emotional Development: The Involvement of Parents” focused on assessing the internal consistency of the measurement instruments used to measure children’s socio-emotional development, levels of social media exposure, and parental involvement practice. Cronbach alpha was employed to determine the reliability of the 53 questionnaire items included in the pilot study. The analysis produced a Cronbach alpha coefficient of 0.927, indicating excellent internal consistency among the items. This coefficient shows that the items are highly correlated and consistently measure the underlying constructs of the study.

## 5. Dependent variable

### 5.1. Socio-emotional development

Socio-emotional development was assessed using an ASQ-2-adapted instrument, which has 15 items measuring children’s emotional regulation, empathy, and social interaction skills. The items focused on the child’s ability to manage emotions, understand others’ feelings, and interact appropriately with peers. Items such as “My child can calm down when upset or frustrated,” “My child shows empathy towards others" and “My child interacts well with peers" were included A composite score was computed by averaging all items, where a higher score indicated better socio-emotional development.

## 6. Independent variables

### 6.1. Social media exposure

Social media exposure was measured using ASQ-2-adapted, which has 18 items assessing the frequency and intensity of children’s engagement with social media and online digital platforms. The items captured the extent of children’s exposure to and interaction with online content in their daily lives. Sample items include “My child spends a lot of time using social media or online video platforms" and “My child is regularly exposed to content from social media or online sources.” The mean of all items was computed to form a composite score, where a higher value indicated greater social media exposure.

## 7. Mediator variable

### 7.1. Parental involvement

Parental involvement was measured using^[22]^ an adaptation of a total of 15 items assessing parental monitoring, communication, and mediation of children’s social media use. The scale captured the degree to which parent supervise, guide and engage with their children regarding online activities. Example items included “I monitor and guide my child’s use of social media or online content” and “I discuss online or social media content with my child to help them understand it.” Responses were averaged to generate a composite score, with higher score reflecting greater parental involvement.

## 8. Analytic approach

**Figure 1.**
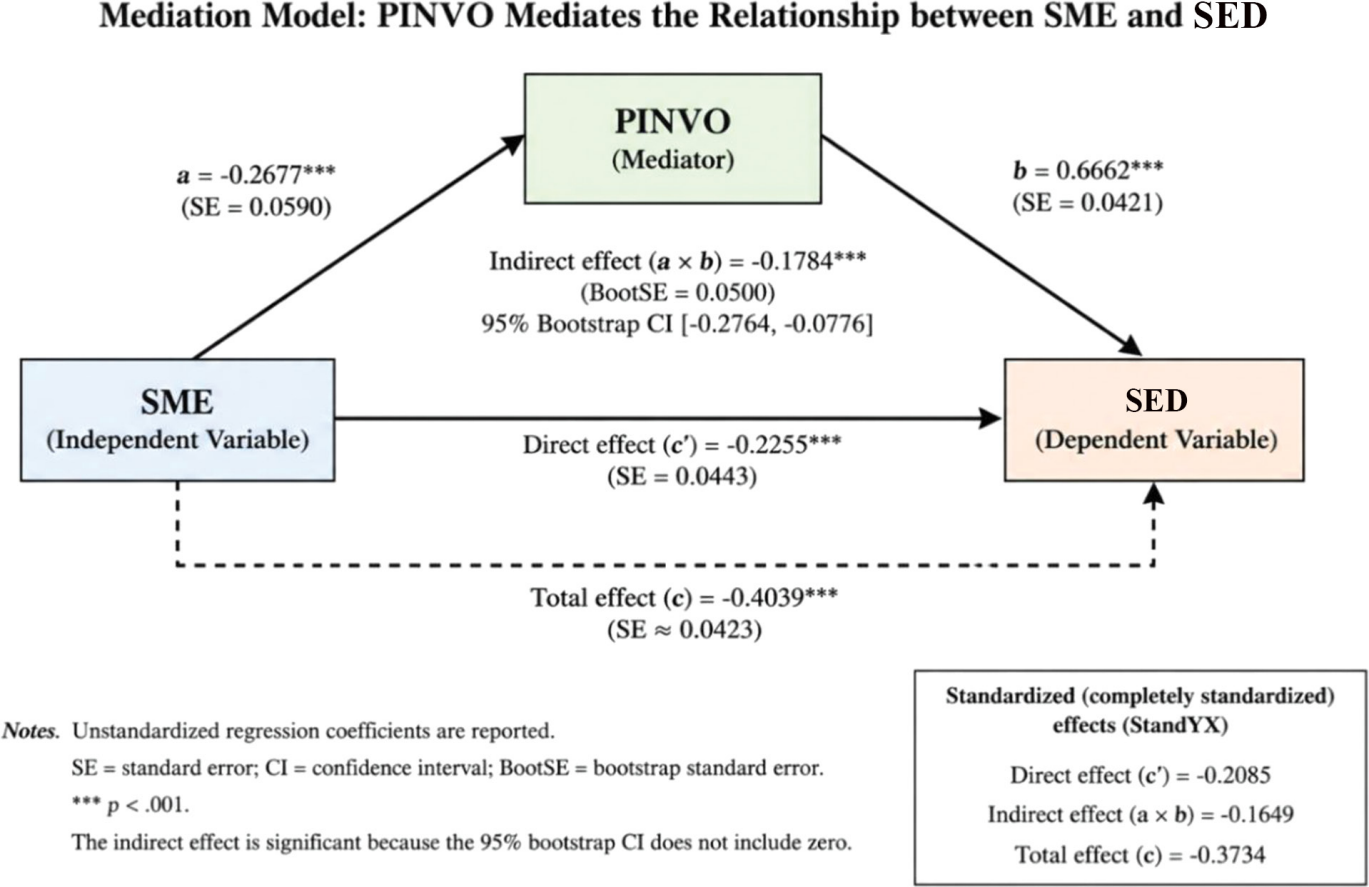
Mediation model of parental involvement in the relationship between social media exposure and socio-emotional development. Unstandardised regression coefficients from the mediation analysis are presented. Solid arrows indicate the estimated direct pathways, and the dashed arrow represents the total effect before the inclusion of the mediator. Values on the paths are regression coefficients, with standard errors (SE) shown in parentheses. The indirect effect was estimated using bootstrap resampling with a 95% confidence interval (CI). Statistical significance is indicated by **P < .001**. PINVO = parental involvement, SED = socio-emotional development, SME = social media exposure.

A mediation analysis was conducted using the PROCESS macro for mediation, moderation, and Conditional Process Analysis Model 4 developed by^[23]^ to examine whether parental involvement mediated the relationship between social media exposure and children’s socio-emotional development. Mediation analysis is important to determine whether the influence of an independent variable on a dependent variable occurs directly or indirectly through another variable. In this study, social media exposure was treated as the independent variable, socio-emotional development as the dependent variable, and parental involvement as the mediator variable. The analysis aimed to determine whether parental involvement played a significant role in explaining how social media exposure affected children’s socio-emotional development. Figure 1 shows the result of the mediation model.

## 9. Results

Social media exposure had a mean score of 2.57 (standard deviation [SD] = 0.97), suggesting that children’s social media exposure to content was comparatively moderate.

All variables shared the same minimum (1.00) and maximum (5.00) values, confirming that the data were correctly coded according to the 5-point Likert scale and that no out-of-range values were present. SDs (≈1.0) indicate acceptable variability in responses, with no evidence of restricted range or abnormal dispersion. Table 3 shows the result of the mean and SD.

**Table 3:** Result of mean and standard deviation.

Variable	Mean	Standard deviation	Min	Max
Socio-emotional development	3.30	1.05	1.00	5.00
Social media exposure	2.57	0.97	1.00	5.00
Parental involvement	3.12	1.02	1.00	5.00

Data analysis reveals that social media exposure significantly predicts parental involvement (β = −0.268, *P* < .001). The negative regression coefficient indicates that increased exposure to social media was associated with lower levels of parental involvement. As children spend more time engaging with social media platforms and digital content, parents may become less involved in monitoring, guiding, or interacting with their children regarding online activities. The results may also imply that excessive engagement with social media reduces opportunities for parent–child interaction and communication. Since parental involvement is an essential factor in children’s emotional and social growth, reduced parental engagement may create challenges in children’s behavioral adjustment, emotional regulation, and interpersonal relationships.

The findings further showed that parental involvement mediated children’s socio-emotional development (β = 0.666, *P* < .001). The positive regression coefficient indicates that higher parental involvement contributes positively to children’s socio-emotional development. This means that children whose parents are more actively involved in their daily lives, communication, guidance, supervision, and emotional support are more likely to demonstrate positive socio-emotional behaviors. Such children may have better emotional control, improved social interaction skills, greater empathy, and healthy relationships with peers and adults. The results emphasize the importance of parental participation in children’s developmental process, particularly in helping children cope with emotional challenges and social pressures associated with the modern digital environment.

The analysis also indicated that social media exposure had a significant direct effect on socio-emotional development (β = −0.226, *P* < .001). This negative relationship suggests that a higher level of social media exposure directly reduces children’s positive socio-emotional development. The result implies that excessive exposure to social media may negatively influence children emotional well-being, social behavior and interpersonal communication. Children who spent extended periods on social media may experience reduced face-to-face interaction, emotional dependency on digital communication, difficulty managing emotions, and limited opportunities for developing social skills through real-life experience.

The mediation analysis further examined the indirect effect of social media exposure on socio-emotional development through parental involvement. The results showed that the indirect effect was statistically significant (β = −0.178, BootSE = 0.050), and the 95% bootstrap confidence interval ranged from −0.276 to −0.078. Since the confidence interval did not include zero, the mediation effect was considered statistically significant. This finding confirms that parental involvement serves as a mediator factor in the relationship between social media exposure and socio-emotional development. In other words, social media exposure negatively affects parental involvement, which in turn negatively influences children’s socio-emotional development.

The result specifically indicates the presence of partial mediation. Partial mediation occurs when both the direct effect and the indirect effect remain statistically significant. In this study, social media exposure continued to have a direct negative effect on socio-emotional development even after parental involvement was included in the model. At the same time, the indirect pathway through parental involvement was also significant. This means that parental involvement explained part (44.2%), but not all, of the relationship between social media exposure and socio-emotional development. Therefore, social media exposure affects socio-emotional development both directly and indirectly through its influence on parental involvement.

The findings suggest that parental involvement plays a protective role in reducing the negative impact of social media exposure on children’s socio-emotional development. Parents who actively monitor and guide their children’s social media use may help children develop healthy emotional response and more positive social behaviour. Active parental involvement may include setting rule regarding screen time, discussing online experiences with children, encouraging balanced activity, and providing emotional support. Such practice may reduce the harmful effect associated with excessive or inappropriate social media use and promote healthy developmental outcomes.

Moreover, the findings highlight the importance of maintaining strong parent–child relationships in the digital age. As social media becomes increasingly integrated into children’s daily lives, parents need to remain engaged in understanding the digital environment their children interact with. Effective parental involvement can help children critically evaluate online content, manage emotional reactions, and maintain healthy social relationships. Parents who communicate openly with their children about social media use may also foster trust and emotional security, which are important components of socio-emotional development.

The results of this study are consistent with existing literature suggesting that parental involvement significantly influences children’s developmental outcomes. The study conducted by^[24]^ has emphasised that supportive parenting practices contribute positively to emotional adjustment, social competence, and behavioural regulation among children. Similarly, social media exposure has been reported to show that excessive use of digital platforms may negatively affect children’s emotional and social well-being. The present finding extends this understanding by demonstrating that parental involvement partially mediates the relationship between social media exposure and socio-emotional development.

The findings also imply that interventions aimed at improving children’s socio-emotional development should not focus solely on reducing social media exposure but should also strengthen parental involvement.

The mediation analysis demonstrated that parental involvement is a significant mechanism through which social media exposure influences children’s socio-emotional development. Social media exposure negatively affects socio-emotional development both directly and indirectly by decreasing parental involvement. The findings underscore the critical role of parents in supporting children’s emotional and social well-being in increasingly digital environments. Strengthening parental involvement may therefore help minimise the adverse effects associated with social media exposure and promote healthier socio-emotional development among children.

## 10. Discussion

The present study examined the mediating role of parental involvement in the relationship between social media exposure and children’s socio-emotional development. The findings revealed that social media exposure negatively predicted parental involvement; parental involvement positively predicted socio-emotional development; and social media exposure negatively affected socio-emotional development both directly and indirectly through parental involvement. The mediation analysis further confirmed that parental involvement partially mediated the relationship between social media exposure and socio-emotional development. These findings provide important insights into how children’s increasing engagement with social media may influence their emotional and social well-being and the important protective role played by parents.

The finding that social media exposure negatively predicts socio-emotional development is consistent with previous studies that reported excessive social media use may negatively influence children’s emotional regulation, social interaction, and behavioural adjustment. This is also confirmed by^[24]^ in China. Existing literature suggests that prolonged exposure to social media may reduce face-to-face communication, increase emotional dependency on digital interactions, and limit opportunities for real-life social experience.^[25]^ Children who spend excessive time on social media platforms may become more vulnerable to emotional instability, anxiety, loneliness, and poor interpersonal relationships. Researchers have also noted that exposure to inappropriate online content, cyberbullying, and unrealistic social comparisons may negatively affect children’s emotional well-being and self-esteem.^[26,27]^ The current finding therefore supports earlier evidence indicating that uncontrolled or excessive social media exposure can create challenges for healthy socio-emotional development among children.

The present study also found that social media exposure significantly reduced parental involvement. This finding aligns with previous research suggesting that increased digital engagement may weaken parent–child interaction and communication.^[28]^ As children spend more time using smartphones, social networking platform and online entertainment, parents may have fewer opportunities to participate in shared activities, emotional conversation, and direct supervision. Existing studies have emphasised that excessive technology use may disrupt family relationship by reducing the quality of parent–child communication and limiting emotional closeness within the family environment. In some cases, parents may also struggle to monitor children’s online activities due to limited digital literacy, busy schedules, or lack of awareness regarding online risk. The current study, therefore, supports the view that a high level of social media exposure may contribute to reduced parental engagement in children’s daily live.

Another important finding of this study is that parental involvement positively predicts socio-emotional development. This result is strongly supported by John Bowlby’s attachment theory and a study conducted by^[29]^ highlight the importance of parents in children’s emotional and social growth. According to attachment theory, positive parent–child relationships provide emotional security and support that help children develop self-confidence, empathy, emotional control, and social competence. Parents who actively communicate with their children, provide emotional guidance, and monitor their activities create a supportive environment that promotes healthy socio-emotional development. Similarly^[30]^ reported that parental warmth, supervision, and involvement are associated with improved emotional regulation, positive peer relationships, and reduced behavioral problems among children.

The findings of this study further demonstrate that parental involvement partially mediates the relationship between social media exposure and socio-emotional development. This means that social media exposure influences socio-emotional development not only directly but also indirectly through its effect on parental involvement.^[28]^ Support mediating relationship by emphasising that parents play an important role in shaping how children interact with digital media. A study on parental mediation theory^[31]^ suggests that parents who actively supervise and guide children’s media use can reduce exposure to harmful content and encourage healthy online behavior. Active parental mediation may involve setting screen-time limits, discussing online experiences, monitoring digital activity, and encouraging balanced offline interaction. Such practices may reduce the negative emotional and social consequences associated with excessive social media use.

The finding of partial mediation indicates that parental involvement explains only part of the relationship between social media exposure and socio-emotional development. This suggests that other factors may also contribute to the influence of social media on children’s socio-emotional outcomes. Previous research has identified several additional factors such as peer influence, type of online content, individual personality traits, school environment, and digital literacy as possible contributors to children’s emotional and social adjustment.^[32]^ For example, educational and age-appropriate digital content may have positive developmental effects, while violent or inappropriate content may contribute to emotional and behavioural difficulties. Similarly, supportive peer relationships and healthy school environments may reduce some of the negative effects associated with social media exposure.

The current findings are also consistent with the ecological systems theory of Bronfenbrenner,^[33]^ which explains that children’s development is influenced by interactions between multiple environmental systems, including family, school, peers, and media environments. Social media has become an important part of children’s social environment, influencing their thoughts, behaviours, and relationships. From an ecological systems perspective, the mesosystem highlights the interaction between family and media environments. Parental involvement operates within this system by regulating and interpreting children’s media experiences thereby mediating the impact of social media exposure on socio-emotional development. However, parents remain one of the most influential factors in shaping children’s developmental experiences. The present study demonstrates that parental involvement continues to play a critical protective role even in increasingly digital environments. Children who receive parental guidance and emotional support may be better equipped to manage online experiences and maintain healthy socio-emotional functioning.

The findings have important practical implications for parents, educators, and policymakers. Parents need to become more actively involved in children’s social media use by maintaining open communication, monitoring children’s online activities, and encouraging a balanced daily routine. Rather than completely restricting digital technology, parents may help children develop responsible and healthy social media habits. Schools may also contribute by educating both parents and children about digital safety, emotional well-being, and responsible technology use. Community-based awareness programmes may further help parents understand the potential risks and benefits associated with children’s social media exposure.

Another important finding of this study is that parental involvement positively predicts children’s socio-emotional development. This finding can be explained by social learning theory, proposed by Albert Bandura, which emphasises that children learn behaviour, emotion, and social skills through observation, imitation, and interaction with parents. When parents actively communicate, provide emotional guidance, and monitor their children, they serve as positive role models that foster self-regulation, empathy, and social competence. Consistent with this view, prior study^[29,30]^ confirm that parental warmth and supervision enhance children’s emotional and behavioural adjustment. The mediating role of parental involvement in the relationship between social media exposure and socio-emotional development can also be explained through social learning theory. Children often model behaviour and emotional responses observed both in their digital environment and from their parents. Thus, while social media may expose children to content that influences emotional and social behaviour, parental involvement can guide the interpretation of such content, regulate usage, and reinforce appropriate behaviour.

The finding additionally suggests the need for interventions aimed at strengthening parental involvement in the digital age. Parenting programmes that focus on digital parenting skills, online supervision strategies, and effective communication techniques may help parents better support their children’s socio-emotional development. Policymakers and child development professionals may also collaborate to promote child-friendly digital environments and encourage responsible social media practices among families.

Generally, the findings of this study support existing knowledge that social media exposure can negatively affect children’s socio-emotional development, while parental involvement serves as an important protective factor. The mediation effect identified in this study highlights the significant role parents play in reducing the harmful effects associated with excessive social media use. Although social media exposure directly affects socio-emotional development, strong parental involvement may help children develop healthy emotional responses, positive social behavior, and improved interpersonal relationships. The study therefore emphasizes the importance of maintaining active parental engagement to support children’s socio-emotional well-being in modern digital environment.

## 11. Limitations

This study has some limitations. The cross-sectional design does not permit causal inference. In addition, the sample was drawn from a single region in Ethiopia, which may limit the generalisability of the findings. Furthermore, the use of parent-reported measures may have introduced social desirability bias. Future studies should employ a longitudinal design, include more diverse samples, and use multiple informants and observational measures.

## 12. Recommendations

Based on the findings of this study, 3 key recommendations are proposed. First, parents should actively monitor and guide their children’s social media use by establishing age-appropriate screen-time limits using app-based parental control features (e.g., screen-time management, content filtering, and activity monitoring), encouraging open communication about online experiences, and providing consistent emotional support. Strengthening parental involvement may help reduce the negative association between social media exposure and children’s socio-emotional development.

Second, schools in collaboration with local education offices should implement digital literacy programmes tailored to Ethiopian families. This programme should educate parents and children on responsible social media use, online safety, cyberbullying prevention, privacy protection, and healthy digital habits while emphasising the importance of parental engagement in children’s online activity.

Third, policymakers, the Ministry of Education, and child-focused organisations should support community-based digital parenting initiatives by providing practical training on effective monitoring strategies, age-appropriate media use, and positive parent–child communication about digital technology. This initiative should be adapted to the cultural and technological context of Ethiopian families to improve its accessibility and effectiveness.

Future research should investigate additional factors influencing children’s socio-emotional development, such as parenting style, peer influence and the type of online content accessed. A longitudinal study is also recommended to establish a causal relationship and examine the long-term effect of social media exposure across different age groups and settings.

## Author contributions

**Writing – original draft:** Tiruye Abdie Hussein, Wohabie Birhan Bitew, Tiruwork Tamiru Tolla.

**Writing – review & editing:** Tiruye Abdie Hussein, Wohabie Birhan Bitew, Tiruwork Tamiru Tolla.
